# Haplotype diversity in mitochondrial DNA reveals the multiple origins of Tibetan horse

**DOI:** 10.1371/journal.pone.0201564

**Published:** 2018-07-27

**Authors:** Lin Yang, Xiaoyan Kong, Shuli Yang, Xinxing Dong, Jianfa Yang, Xiao Gou, Hao Zhang

**Affiliations:** 1 Key Laboratory of Animal Genetics, Breeding and Reproduction, Ministry of Agriculture, College of Animal Science and Technology, China Agricultural University, Beijing, China; 2 College of Animal Science and Technology, Yunnan Agricultural University, Kunming, China; Banaras Hindu University, INDIA

## Abstract

The Tibetan horse is a species endemic to the Tibetan plateau, with considerable economic value in the region. However, we currently have little genetic evidence to verify whether the breed originated in Tibet or if it entered the area via an ancient migratory route. In the present study, we analyzed the hypervariable segment I sequences of mitochondrial DNA (mtDNA) in 2,050 horses, including 290 individuals from five Tibetan populations and 1,760 from other areas across Asia. Network analysis revealed multiple maternal lineages in the Tibetan horse. Component analysis of sub-lineage F3 indicated that it decreased in frequency from east to west, a trend reflected both southward and northward from Inner Mongolia. Analysis of population genetics showed that the Deqen horse of eastern Tibet was more closely related to the Ningqiang horse of northern China than to other Tibetan horses or the Yunnan horse. These results indicated that the Tibetan horse migrated first from Central Asia to Mongolia, moved south to eastern Tibet (near Deqen), then finally westward to other regions of Tibet. We also identified a novel lineage K that mainly comprises Tibetan and Yunnan horses, suggesting autochthonous domesticated origin for some Tibetan horse breeds from local wild horses. In conclusion, our study demonstrated that modern Tibetan horse breeds originated from the introgression of local wild horses with exotic domesticated populations outside China.

## Introduction

Tibetan horses currently number at over 270,000 individuals[1] and are mainly distributed in the Qinghai-Tibet Plateau, the highest ecosystem in the world (average elevation >4,000 m). With outstanding adaptability to extreme, high-altitude environments, the Tibetan horse has historically been of high economic value for Tibetans, playing a critical role in transportation, stock farming, and trade (e.g., horse-silk trade with Han Chinese during the Tang and Song dynasties, 618~1279 AD). Archaeological records indicate that horses have been present in Tibet at least since the Neolithic period over 4,000 years ago. Generally, indigenous domestic animals in Tibet are thought to have been introduced with the migration of Tibetan ancestors from northern China approximately 7,000 years ago [2, 3]. Research based on mitochondrial DNA (mtDNA) revealed that Tibetan mastiff [4], Tibetan sheep [5] and Tibetan chicken [6] were derived from outside of Tibet, and Tibetan yak [7] and Tibetan pig [8] were appear to have a local origin. However, we currently have no direct genetic evidence regarding whether Tibetan horses originated in the region or were descended from exotic breeds.

The earliest horse domestication is widely accepted to have occurred on the Eurasian steppes of Kazakhstan between the fifth and fourth millennium BC[9]. Furthermore, as they spread out from their original range, domestic horses were repeatedly restocked with local wild horses[10], and multiple domestication events or introgressions of wild females were thought to have occurred [11–17]. Thus, most modern horses have several maternal origins that can be divided into seven major lineages (A~G) and 19 clusters [13–16, 18]. Lineage F is more common in Eastern populations, supporting an ancestral line that entered the domestic horse gene pool from East Asia [13, 14]. Existing Chinese domestic horses are grouped into lineage F and a new lineage H in the southwest, suggesting a combined origin of horses introduced from outside of China and local wild horses [19]. However, it is not clear whether this conclusion applies to Tibetan horses.

A previous study using seven mtDNA sequences tentatively concluded that Tibetan horses have multiple maternal lineages, similar to other horse breeds [20]. To verify this outcome, here we performed a comparative mtDNA analysis of the Tibetan horse with relevant populations across Asia. Our results should elucidate the origin of this ancient breed, clarifying the degree and nature of introgression from exotic horses.

## Materials and methods

### Sample collection and sequencing

**Fig 1:**
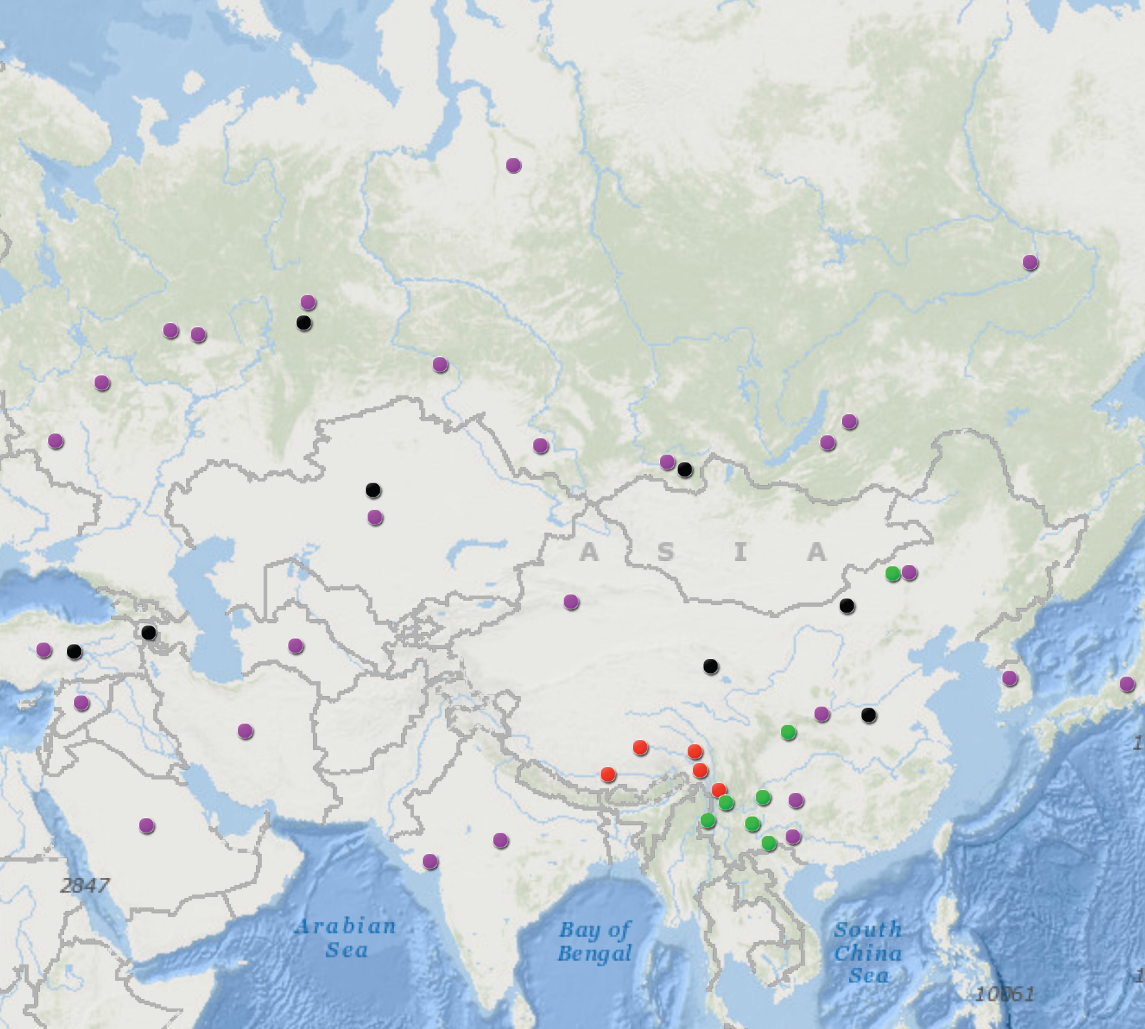
Geographic distribution of the total 2,050 samples. In the map, red dots represent Tibetan horses; green dots represent Yunnan horses and northern China horses; purple dots represent other modern horses and black dots represent ancient horses.

Blood samples were collected from 721 horses of the Tibetan highland (n = 272), Yunnan province (n = 387), and northern China (n = 62). Five populations were represented from Tibetan highland: Xigazê (XGZ, n = 49; 29°16′N, 88°52′E), Nagqu (NQ-T, n = 41; 31°28′N, 92°03′E), Qamdo (QD, n = 52; 31°08′N, 97°10′E), Zogang (ZG, n = 45; 29°40′N, 97°50′E), and Deqen (DQ, n = 85; 27°49′N, 99°42′E). Likewise, Yunnan samples included five populations: Lijiang (LJ, n = 157; 26°51′N, 100°13′E), Tengchong (TC, n = 52; 25°01′N, 98°29′E), Kunming (KM, n = 14; 24°52′N, 102°49′E), Zhaotong (ZT, n = 35; 27°20′N, 103°42′E), and Wenshan (WS, n = 129; 23°23′N, 104°13′E). Finally, two populations represented the northern China region: Ningqiang (NQ, n = 26; 32°29′N, 106°15′E) and Inner Mongolia (MG, n = 36; 43°55′N, 116°02′E). The experiments were approved by the Animal Welfare Committee of the State Key Laboratory for Agro-biotechnology of the China Agricultural University (Approval number XK257). Genomic DNA was isolated following standard phenol/chloroform extraction methods. Based on the complete horse mtDNA sequence, a primer set (5′-^15359^TGT AAA CCA GAA AAG GGG GAA AAC^15382^-3′, 5′-^16059^TTG CTG ATG CGG AGG AAT AAC G^16080^-3′) was designed to amplify a 722 bp segment of the mtDNA control region, hypervariable segment I (HVI). Sanger sequencing was employed to sequence PCR amplicons. For comparison, 1,329 mtDNA HVI sequences from horses across Asia (1,235 domestic horses and 94 ancient horses) were downloaded from Genbank (https://www.ncbi.nlm.nih.gov/genbank/). S1 Table lists all 2,050 sequences used in this study, and their geographic distribution is shown in Fig 1.

### Sequence alignment and sequence variation

Available mtDNA sequences were trimmed to be as long as possible for improved accuracy in genetic analysis. A 421 base-pair (bp) segment that was the hypervariable segment I region of mtDNA was trimmed from 15436–15856 bp (Genbank accession no. X79547) for all 721 sequenced horses in this study to analyze genetic diversity. The accession numbers of 721 sequences had been uploaded Genbank (MH032886~MH033606). Additionally, a 247 bp (15494–15740 bp) segment that was used to study maternal origin of horse in many studies [13–16] was trimmed for all 2,050 sequences to examine evolutionary relationships. Sequences were aligned in Muscle 3.8.31[21] on the Linux virtual server, then trimmed and edited in BioEdit (http://www.mbio.ncsu.edu/bioedit/bioedit.html). The number and proportion of substitutions, transition/transversion ratio, number of haplotypes, nucleotide diversity (Pi), and haplotype diversity (h) were calculated in DnaSP v.5.1[22]. The major maternal lineages are defined by specific diagnostic mutations. Component analysis for lineage frequencies in each breed or population was estimated through counting.

### Phylogenetic analysis

Network 4.0 (http://www.fluxus-engineering.com) was used to construct median joining networks for phylogenetic analysis of all haplotypes. Default parameter settings were used, except for the following. Star-like phylogenetic clusters were contracted into one ancestral type, epsilon (0) was chosen, weights of mutational hotspot site (15650) identified from previous studies[16, 18] and mutational hotspot site (15600) from the present work were set to zero, and haplotypes over 2% were shown in final networks[19]. Bayesian consensus trees using Monte Carlo Markov chains (MCMC) were also constructed in MrBayes 3.1[23] to infer the evolutionary relationship among haplotypes. Two independent runs were performed, each with two million generations, starting from a random tree. The sampling frequency was set to 1/1,000 with the first 20% tree discarded as burn-in. Average standard deviation of split frequencies was <0.05 to ensure clustering quality.

### Detection of genetic relationships among horse populations

Fifteen horse populations (12 populations in this study, plus populations from Central Asia; Guizhou, 26°36′N, 106°42′E; and Guangxi, 23°54′N, 106°36′E) were used to analyze genetic relationships across geographical distribution. Small or geographically close populations were merged to ensure reliability of the results. For example, the NQ population included horses from Ningqiang, Guanzhong, and Guan mountain. Pairwise mean distances among the 15 populations were calculated using the distance option in MEGA 7.0[24], with a bootstrap value of 1,000. Based on the resultant pairwise population mean distances matrix, a neighbor-joining phylogenetic tree was then constructed by Phylip 3.695[25].

## Results

### Genetic diversity of Tibetan horses

We detected 91 haplotypes with 57 polymorphic sites in 272 Tibetan horses (S2 Table). The variable sites included 53 transitions (A/G and T/C) and four transversions (A/T). Maximum and minimum haplotype counts were 33 in Xigazê and 21 in Zogang, respectively. In Tibetan horses, nucleotide diversity ranged from 0.01798 ± 0.00113 in Qamdo to 0.02266 ± 0.00073 in Xigazê, while haplotype diversity (h) ranged from 0.920 ± 0.025 in Zogang to 0.977 ± 0.010 in Xigazê, suggesting considerable genetic diversity in Tibetan horse populations (Table 1). Overall, genetic diversity was highest in northern China horses, followed by Tibetan horses and then Yunnan horses, although nucleotide and haplotype diversity in the latter two populations were also relatively high.

**Table 1:** Genetic diversity of Chinese domestic horses based on a 421 bp fragment of hypervariable segment I in mitochondrial DNA.

Geographic grouping	Populations	n/N	Pi	h
Tibetan horses	Nagqu	24/41	0.01994±0.00106	0.970±0.011
	Xigazê	33/49	0.02266±0.00073	0.977±0.010
	Qamdo	30/52	0.01798±0.00113	0.968±0.012
	Deqen	30/85	0.01851±0.00083	0.948±0.013
	Zogang	21/45	0.01919±0.00095	0.920±0.025
	overall	91/272	0.02020±0.00043	0.977±0.003
Yunnan horses	Lijiang	53/157	0.01935±0.00060	0.941±0.011
	Tengchong	22/52	0.01937±0.00101	0.943±0.016
	Kunming	8/14	0.01942±0.00160	0.901±0.058
	Wenshan	27/129	0.01772±0.00064	0.909±0.016
	Zhaotong	8/35	0.02172±0.00145	0.850±0.025
	overall	72/387	0.01915±0.00040	0.932±0.008
Northern China horses	Mongolia	32/36	0.02318±0.00115	0.992±0.009
	Ningqiang	18/26	0.02038±0.00174	0.957±0.025
	overall	47/62	0.02281±0.00087	0.988±0.006

n, number of haplotypes; N, sample size per population; Pi, nucleotide diversity; h, haplotype diversity.

### Phylogenetic analysis of Chinese horses

**Fig 2:**
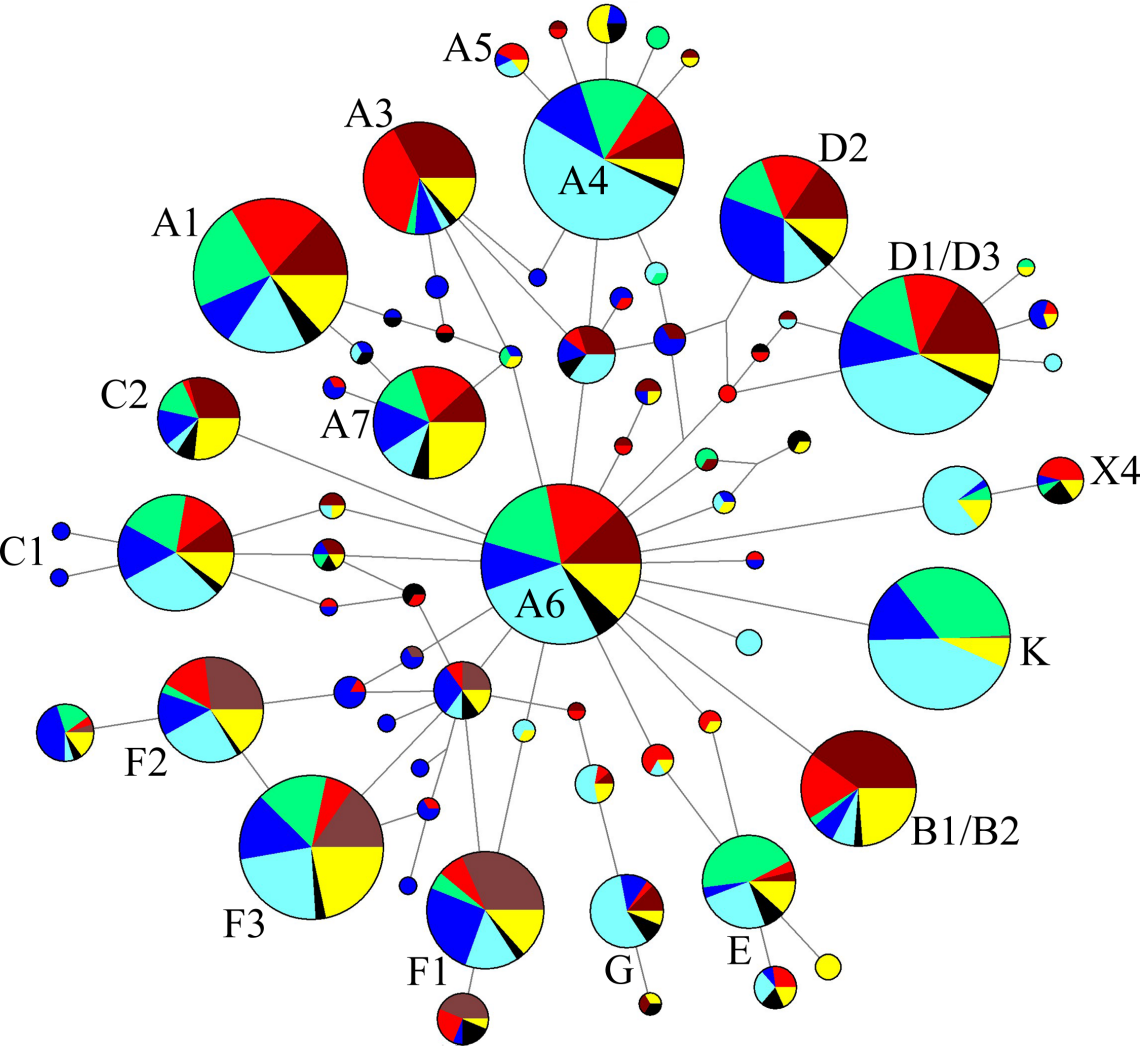
Median joining network was constructed based on 262 haplotypes in 2,050 mtDNA sequences. Circles are proportional to the number of horses represented. Colors of circular sector indicate horses in different areas, for example, brown, Middle East; red, Central Asia including Northeast Asia, Northern Asia and Northwest Asia; green, Tibetan highland; deep blue, northern China including Inner Mongolia, Ningqiang, Guan mountain, Guanzhong; light blue, Chinese south horses including Yunnan, Guizhou and Guangxi; black, ancient horses in Asia; yellow, horses including Japan, Korea, India, Przewalskii horses and unnamed Chinese horses.

Across 2,050 individuals, we found 262 haplotypes (S3 Table) for constructing a phylogenetic network (Fig 2). Nine major lineages (A–G, X4, and K) were identified. Eight lineages (A–G and X4) were named based on previous studies[15, 16, 18], while K was a novel lineage named in this study (S4 Table). Tibetan horses were in most of these lineages except G (Fig 2 and S3 Table).

Lineage K including three haplotypes was almost entirely composed of Chinese indigenous horses (98.3%, 121 individuals) (S3 Table), with 72.7% being Tibetan and Yunnan horses. Mirroring the genetics, most lineage K horses were distributed along the border of Yunnan and Tibet. Lineage X4[18] could be divided into two sub-lineages both containing many Chinese native horses, especially those from Yunnan.

The Bayesian consensus tree including 262 haplotypes (S1 Fig) was consistent with the phylogenetic network analysis for major lineages. Lineages B, D, E, G, and X4 were clearly separated from lineage A. However, lineages C, F, and K were unexpectedly clustered with lineage A, likely because the diagnostic mutational motifs were similar across these four lineages.

### Lineage F3 in the whole Asia horse populations

Lineage F included many Chinese indigenous horses and can be divided into three sub-lineages: F1, F2, and F3. Respectively, they contained 42.1%, 53.6%, and 55.4% Chinese horses. In F3, we found five ancient horse individuals from Siberia (FJ204322; >4,000 years ago) and Inner Mongolia (FJ204337, DQ900930, EU931606, EU931608; >2,500 years ago). Thus, sub-lineage F3 appeared to have existed at least 4,000 years ago, approximately 1,500 years before the horse's initial domestication in Kazakhstan.

**Fig 3:**
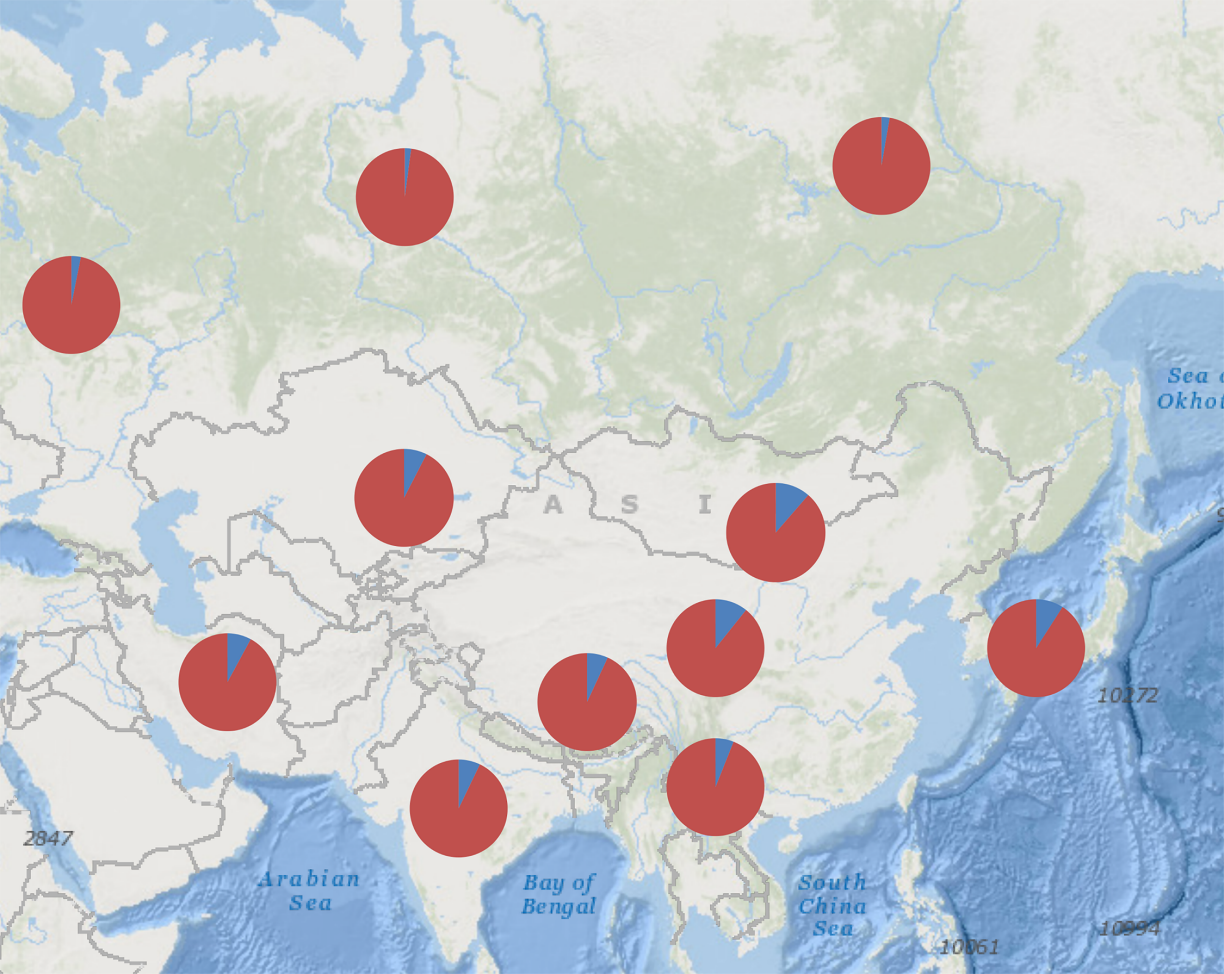
The percentage distribution of sub-lineage F3 in different regions of Asia. In the figure, blue circular sector represents the percentage content of F3.

The component analysis of sub-lineage F3 across Asia (Fig 3) revealed that the sub-lineage gradually increased from west to east, peaking in Inner Mongolia. Additionally, starting from Inner Mongolia, F3 decreased gradually southward and northward. Taken together, our results suggest that F3 likely originated in Inner Mongolia or nearby.

### Lineage F3 and K in Tibetan and Yunnan populations

The component analysis of lineages F3 and K in ten Tibetan and Yunnan populations (Table 2) further indicated that DQ and LJ contained 12.8% and 12.0% F3, respectively, a greater percentage than in the other Tibetan and Yunnan populations. Moreover, among the five Tibetan horse populations, the percentage distribution of lineage K was highest (24.4%) in ZG, followed by QD (23.0%), DQ (10.5%), XGZ (10.2%), and NQ-T (9.5%). Among the five Yunnan horse populations, the percentage distribution of lineage K was highest (13.9%) in LJ, then in WS (10.7%), KM (7.1%), TC (5.8%), and ZT (2.9%).

**Table 2:** Percentage distribution of sub-lineage F3 and K in Tibetan and Yunnan horses.

	Tibetan horses					Yunnan horses					
	XGZ (n = 49)	NQ-T (n = 42)	QD (n = 52)	ZG (n = 45)	DQ (n = 86)	LJ (n = 158)	TC (n = 52)		KM (n = 14)	WS (n = 131)	ZT (n = 35)
F3	5	0	2	0	11	19	3		0	4	0
	10.2%	0.0%	3.8%	0.0%	12.8%	12.0%	5.7%		0.0%	3.1%	0.0%
K	5	4	12	11	9	22	3		1	14	1
	10.2%	9.5%	23.0%	24.4%	10.5%	13.9%	5.8%		7.1%	10.7%	2.9%

XGZ, Xigazê; NQ-T, Nagqu; QD, Qamdo; ZG, Zogang; and DQ, Deqen; LJ, Lijiang; TC, Tengchong; KM, Kunming;ZT, Zhaotong; and WS, Wenshan.

### Genetic relationships among Asian populations

**Fig 4:**
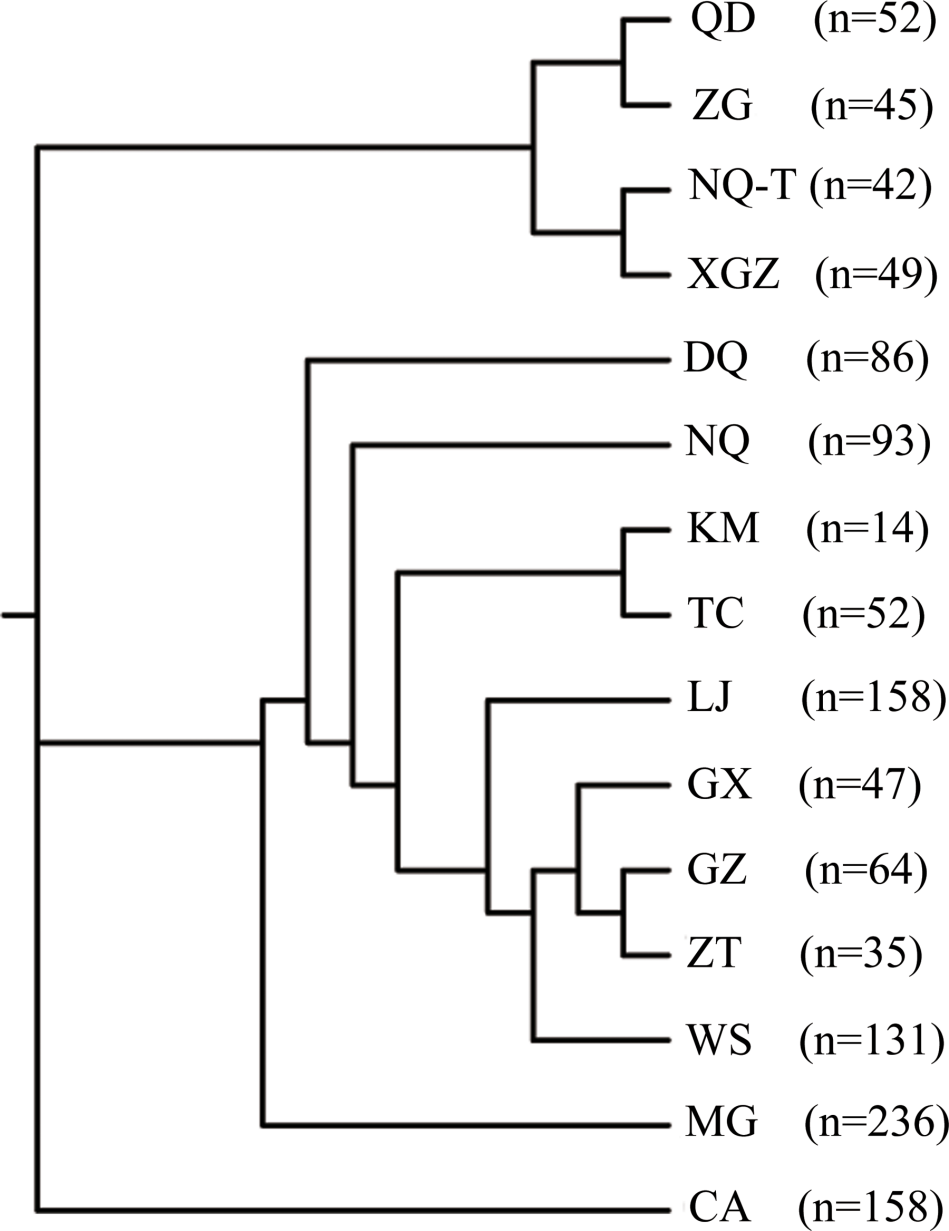
A neighbor joining tree based on 15 horse populations in different area calculated by Phylip. In the figure, abbreviations represent different populations, QD, Qamdo; ZG, Zogang; NQ-T, Nagqu; XGZ, Xigazê; DQ, Deqen; NQ, Ningqiang; KM, Kunming; TC, Tengchong; LJ, Lijiang; GX, Guangxi; GZ, Guizhou; ZT, Zhaotong; WS, Wenshan; MG, Inner Mongolia; CA, Central Asia including Turkmenistan, Kazakhstan, Tuva, Altai, Buryat and Xinjiang Autonomous Region. The numbers in the brackets represent the size of the populations.

Pairwise genetic distances of 15 populations (S5 Table) revealed that the Central Asian and MG horses had genetic distances >0.025, higher than other 13 populations. The neighbor-joining tree indicated that these 15 populations separated into two major clusters (Fig 4). Tibetan horses (QD, ZG, NQ-T, and XGZ) formed their own group, while Yunnan horses (LJ, TC, KM, WS, and ZT) formed a separate group with Guizhou and Guangxi horses. Interestingly, the Tibetan DQ population was more closely related to northern China NQ population and Yunnan horses than to the other four Tibetan populations, possibly because Deqen is at the junction of Tibet and Yunnan.

## Discussion

We found that the five Tibetan horse populations can be clustered into most previously reported lineages, suggesting a complex origin. Most Tibetan haplotypes were present in the originating Kazakhstan lineage, indicating that ancestors of Tibetan horses generally immigrated from other regions. Generally, livestock, such as horse and sheep, were usually migrated along with nomadic tribes as they were important transport tools and food resources for people. In comparison, domestic sheep appear to have undergone two migratory waves[26], spreading in the upper and middle reaches of the Yellow River about 3,000–5,000 years ago in conjunction with the Di-Qiang expansion[27]. Data such as these are extremely useful in helping researchers better understand the demographic forces underlying animal domestication and migration throughout history[28, 29].

Generally, genetic diversity is higher in a domestic species’ originating region, and the frequencies of certain lineages tend to exhibit a geographical gradient as they disperse outwards [13, 30]. In this study, we found that Inner Mongolia was likely the ancestral region of sub-lineage F3, collaborating previous reports suggesting a Far Eastern origin for lineage F overall[13–15, 18]. Furthermore, we found that the percentage of sub-lineage F3 was highest in Deqen (the boundary between Tibet and Yunnan), decreasing gradually westward and southeastward. However, upon reaching Xigazê, F3 percentage was higher than in adjacent areas. This pattern may be due to gene flow or immigration between Xigazê and other Tibetan or Yunnan horse populations through the ancient Tea-Horse Road. This was a trade route from approximately 1,000 years ago that saw the exchange of tea and other commodities from Yunnan for Tibetan horses.

We also observed a closer genetic relationship between Deqen and Ningqiang than between any of the remaining Tibetan horse populations. Additionally, northern China horse had higher genetic diversity than both Tibetan horse and Yunnan horse, suggesting increased gene flow in northern China. These data corroborate the close association between Qamdo and native Mongolian horses[31], likely reflecting introgression from northern horses into Tibetan breeds. Historical records provide some clues regarding the likely path of gene flow. Notably, the South Silk Road of the Tang dynasty (618~907 AD) connected central China with the Tibetan plateau, beginning from Xi’an of Shanxi province, then passing through Gansu, Qinghai, Qamdo, Deqen, westward to Xigazê, and finally reaching Nepal. Furthermore, the Mongolian westward expansion on horseback during the Yuan dynasty likely exerted a direct genetic influence on Tibetan horses. Considering this evidence, we propose that the migratory route of some ancestral Tibetan horses began in Central Asia, moved up to Inner Mongolia, then south to eastern Tibet (Deqen), and finally spread throughout the entire Tibet Autonomous Region.

Lineage K was novel to this study, but is very similar to a previously reported lineage H[19], comprising most indigenous horses of southwest China. Lineage K was mainly composed of Tibetan and Yunnan horses. Similar to our findings for sub-lineage F3, lineage K percentage was highest in ZoGang, gradually decreasing with westward and eastward movement. Low occurrence and restricted geographical distribution of a lineage indicate that its presence likely represents additional introgression into an existing domestic population rather than independent domestication events[26]. Overall, our data suggest that the domestication of Tibetan horses involved introgression from local wild horses.

To conclude, we demonstrated multiple maternal origins in Tibetan horses and provided evidence of an external migratory route into Tibet. Moreover, we found a novel lineage K with restricted geographical distribution (mainly in southwest China). Its presence in Tibetan populations suggests subsequent introgression into ancient local Tibetan horses and thus the latter group’s direct contribution to the domestication process. In summary, our results support an origin for the Tibetan horse that includes introduced breeds from outside of China and introgression from local wild populations.

## Supporting information

S1 Fig A Bayesian consensus tree was constructed using 262 haplotypes. Difference color balls represent different lineages.
(TIF)

S1 Table 2050 Asia horses including 721 Chinese indigenous horses in present study, 1235 domestic horses and 94 ancient horses across Asia retrieved from Genbank. (XLSX)

S2 Table Haplotypes distribution in 272 Tibetan horses based on 421bp (15436~15856). (XLSX)

S3 Table 262 haplotypes in 2050 individuals based on 247bp (15494~15740). (XLSX)

S4 Table Sequence motifs of the mtDNA clusters. (XLSX)

S5 Table Pairwise genetic distance among the 15 horse populations. (XLSX)
