# Supplementary figures and images for: Haplotype diversity in mitochondrial DNA reveals the multiple origins of Tibetan horse
Source: PLoS One. 2018 Jul 27;13(7):e0201564. doi: 10.1371/journal.pone.0201564 (PMC6063445; doi:10.1371/journal.pone.0201564)

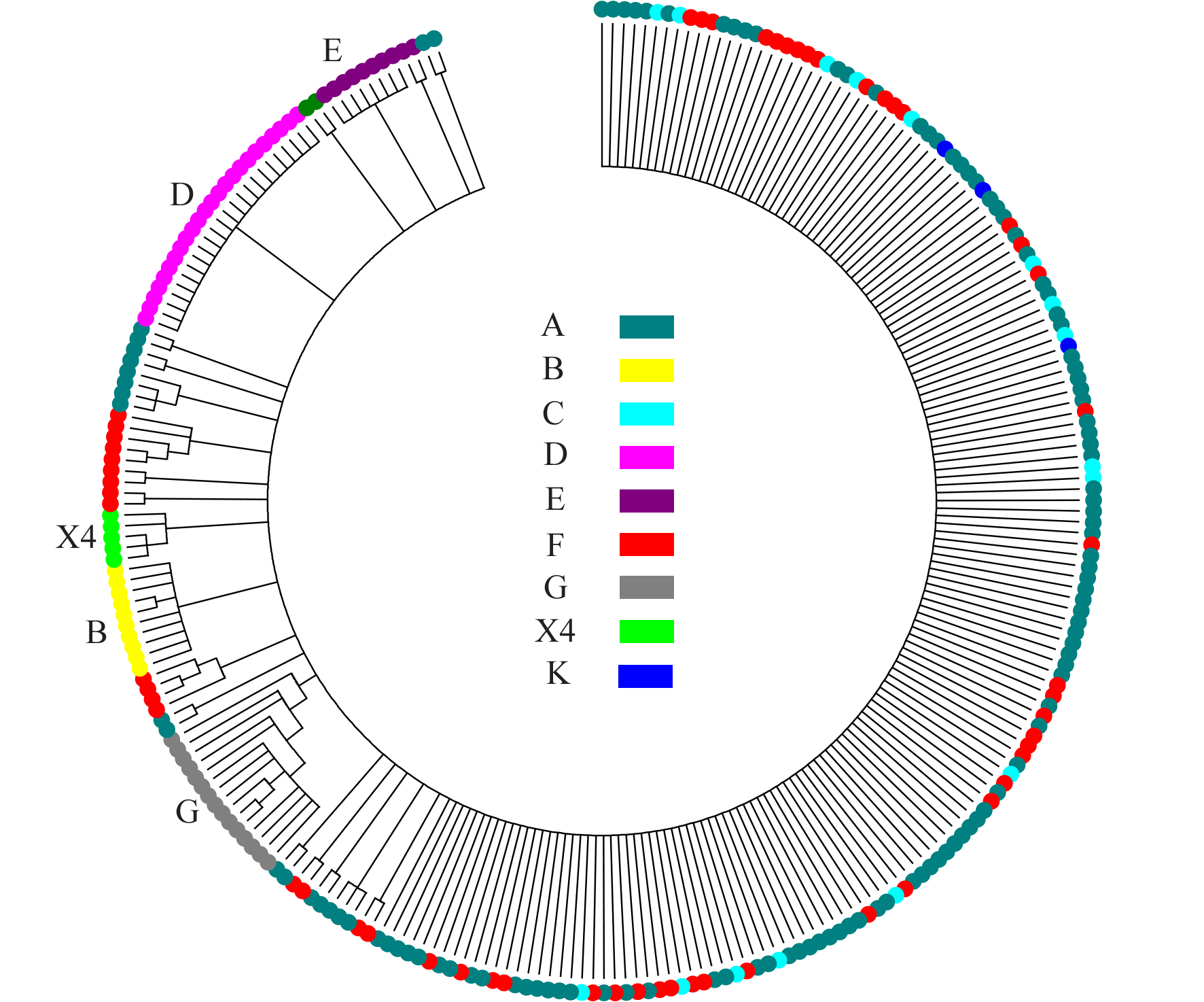

Supplement: S1 Fig — Difference color balls represent different lineages. (TIF) [file pone.0201564.s001.tif]
